# Corrigendum: Daxx inhibits hypoxia-induced lung cancer cell metastasis by suppressing the HIF-1α/HDAC1/Slug axis

**DOI:** 10.1038/ncomms14502

**Published:** 2017-02-07

**Authors:** Ching-Wen Lin, Lu-Kai Wang, Shu-Ping Wang, Yih-Leong Chang, Yi-Ying Wu, Hsuan-Yu Chen, Tzu-Hung Hsiao, Wei-Yun Lai, Hsuan-Hsuan Lu, Ya-Hsuan Chang, Shuenn-Chen Yang, Ming-Wei Lin, Chi-Yuan Chen, Tse-Ming Hong, Pan-Chyr Yang

Nature Communications 7: Article number: 13867; DOI: 10.1038/ncomms13867 (2016); Published 12 22 2016; Updated 02 07 2017

The original version of this Article contained an error in the spelling of the author Yih-Leong Chang, which was incorrectly given as Yi-Liang Chang. This has now been corrected in both the PDF and HTML versions of the Article.

