# Supplementary material for: Daxx inhibits hypoxia-induced lung cancer cell metastasis by suppressing the HIF-1α/HDAC1/Slug axis
Source: Nat Commun. 2016 Dec 22;7:13867. doi: 10.1038/ncomms13867 (PMC5192219; doi:10.1038/ncomms13867)
Supplement: Supplementary Information — Supplementary Figures, Supplementary Tables, Supplementary Methods. [file ncomms13867-s1.pdf]

S1a

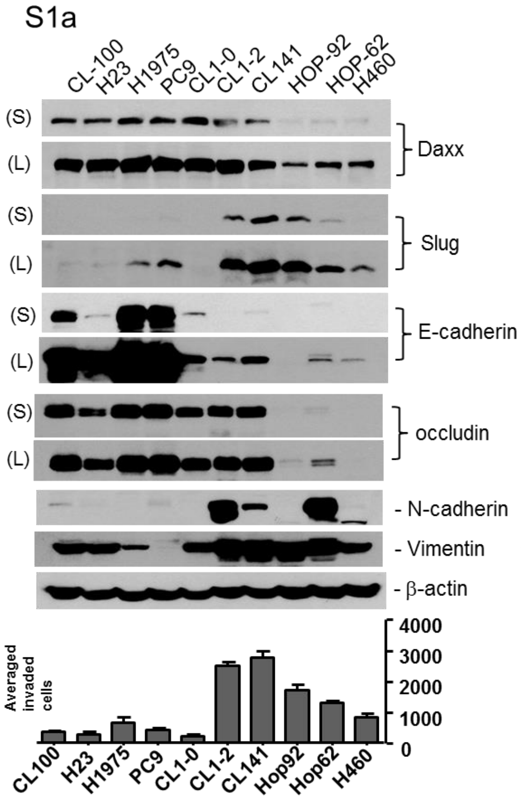

Cell line	Histology	EGFR	Kras
CL100	Squamous cell carcinoma	Del exon19	WT
H23	Adenocarcinoma	WT	mut
H1975	Adenocarcinoma	L858R /T790M	WT
PC9	Adenocarcinoma	Del exon19	WT
CL1-0	Adenocarcinoma	WT	WT
CL1-2	Adenocarcinoma	WT	WT
CL141	Adenocarcinoma	WT	WT
HOP-92	Large-cell carcinoma	WT	WT
HOP-62	Adenocarcinoma	WT	mut
H460	Large-cell carcinoma	WT	mut

S1b

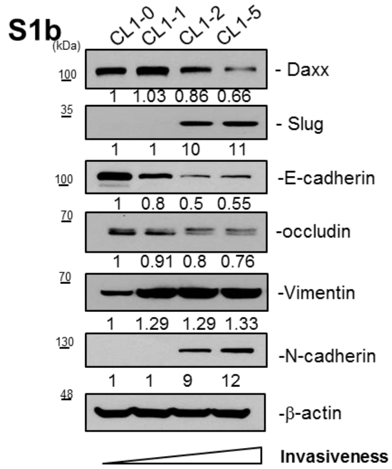

Supplementary Figure 1. The cell lines information. Endogenous protein levels of Daxx, Slug, the epithelial markers E-cadherin and occludin, and the mesenchymal markers vimentin and N-cadherin in NSCLC cell lines **(a)** and CL-series cell lines **(b)**. Right panel indicated the EGFR and K-ras genetic status of each cell line.

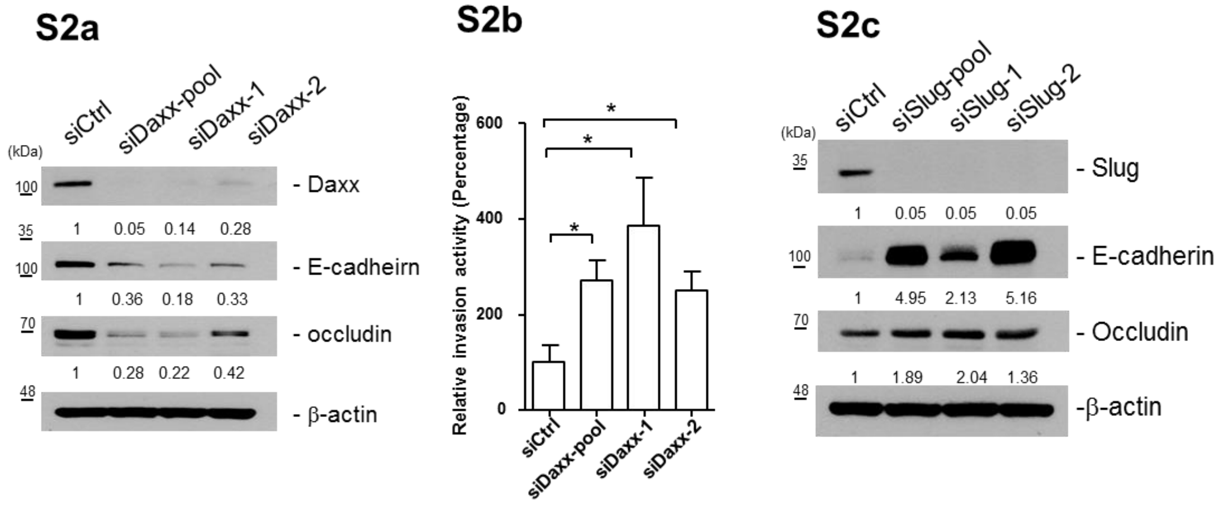

Supplementary Figure 2 Knock-down of Daxx or Slug effected EMT markers and invasiveness. (a) CL1-2 cells transfected with control siRNA (siCtrl), a pooled siRNA (siDaxx-pool) or individual siRNAs (siDaxx-1 and siDaxx-2) were lysed and analyzed by Western blotting. Expression of the indicated proteins was assessed by Western blotting. **(b)** The effects of Daxx silencing in CL1-2 cells were assessed by the Matrigel invasion assay. **(c)** CL1-2 cells were transfected with control siRNA (siCtrl), siSlug-pool, or individual siRNAs (siSlug-1 and siSlug-2) for 48 h. Expression of EMT markers and Slug was detected by Western blotting. Error bars represent s.d. *P<0.05, **P <0.01, paired two-way Student's t-test.

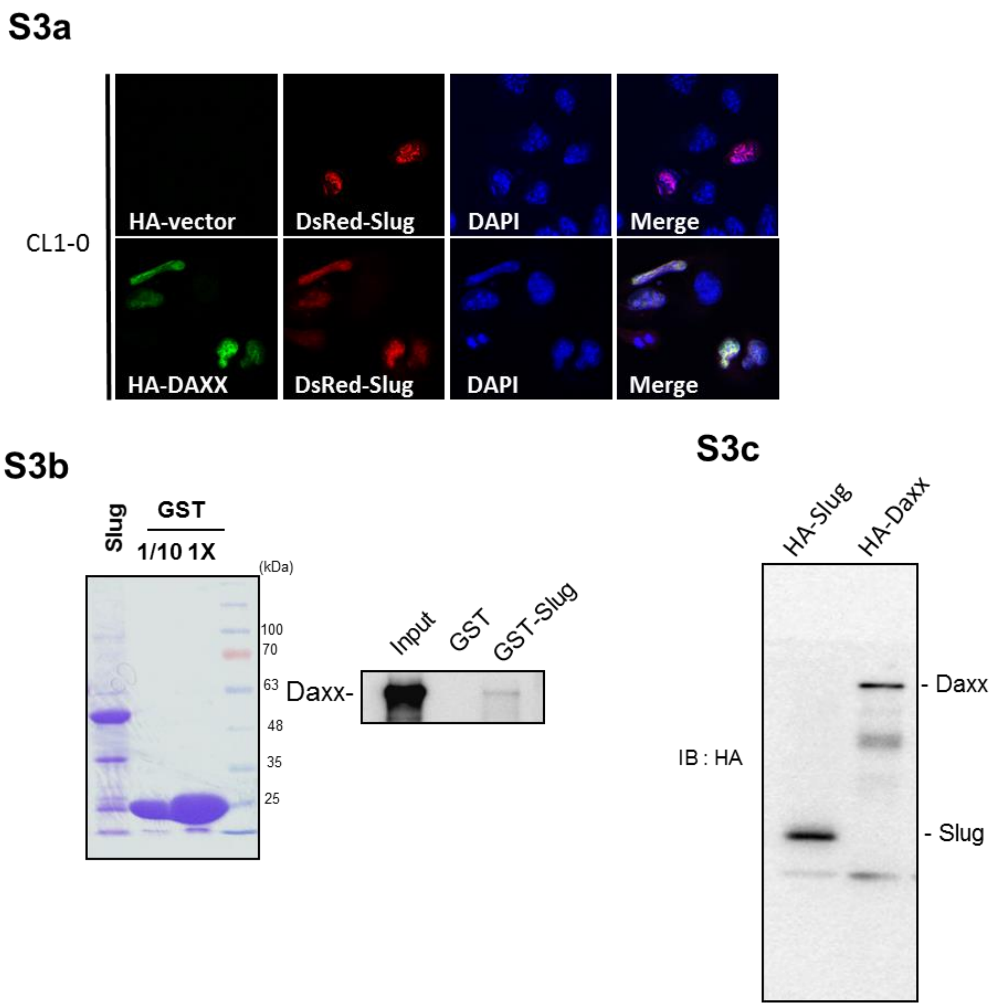

Supplementary Figure 3. Co-localization and interaction of Daxx and Slug. (a) Subcellular localization of Daxx (green), Slug (red), and DAPI (blue) by immunofluorescence staining. CL1-0 cells were co-transfected with HA vector or HA-Daxx plus DsRed-Slug. 36 hours after transfection, cells were fixed and immunostained with an anti-HA or control antibody. **(b)** Protein concentration of GST alone or GST-tagged Slug purified and conjugated with GST-agarose beads, as detected by Coomassie blue staining (left panel). Daxx protein was generated by *in vitro* translation and then incubated with GST-beads or GST-Slug-beads for 1 hr. After washing, protein complexes were determined by immunoblotting. **(c)** Immunoblotting of *In vitro* translation of HA-Daxx and HA-Slug.

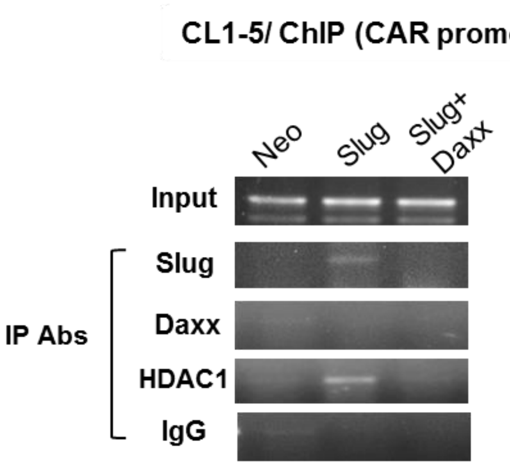

Supplementary Figure 4. Detection of protein recruitment to the CAR promoter region by ChIP assays. Lysates of CL1-5 cells transfected with control vector (Ctrl), Slug, or Slug+ Daxx were used in ChIP assays employing the indicated antibodies. The precipitated CAR promoter region was detected by PCR.

S5a

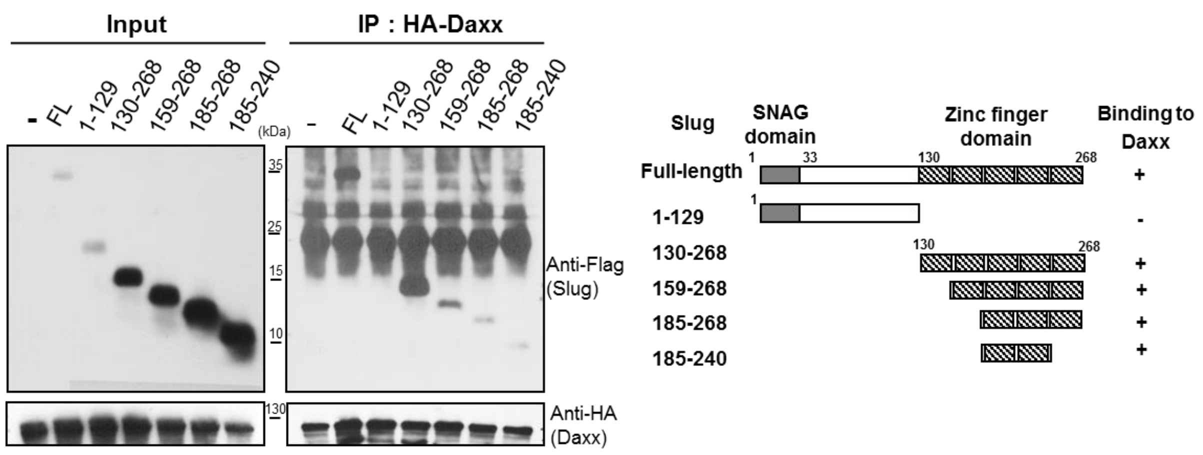

S5b

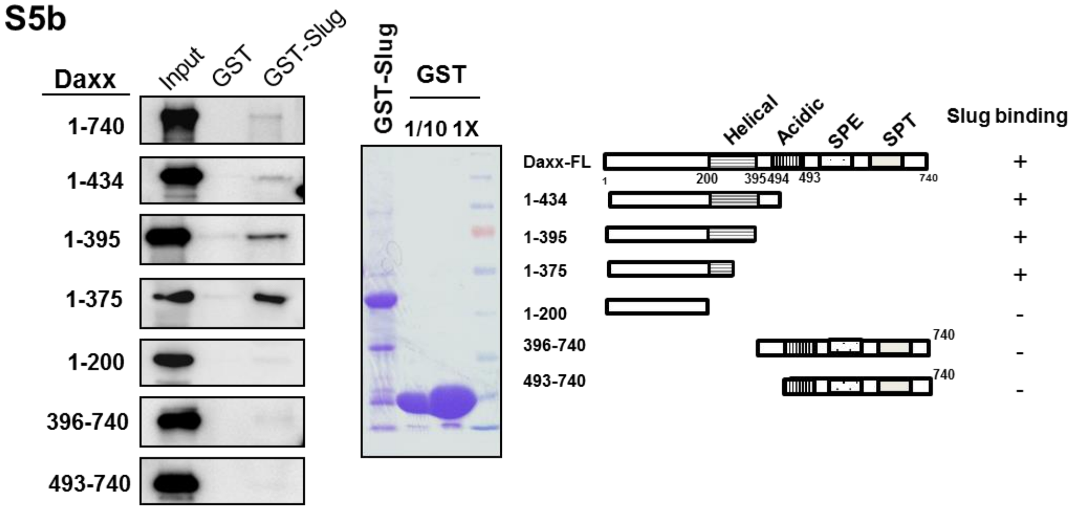

Supplementary Figure 5. Slug-Daxx interactions dissected by immunoprecipitation and GST pull-down assay. (a) H1299 cells transfected with HA-Daxx were co-transfected with different Flag-Slug deletion mutants (or an empty vector) and treated with MG132 for 5 h. Cell lysates were immunoprecipitated (IP) with anti-HA antibodies, and analyzed by immunoblotting (IB). **(b)** The concentrations of GST protein or GST-tagged Slug protein, purified and conjugated with GST-agarose beads, were determined by Coomassie blue staining (upper panel). Daxx protein generated by *in vitro* translation was then incubated with GST beads or GST-Slug beads for 1 h. After washing, protein complexes were determined by immunoblotting.

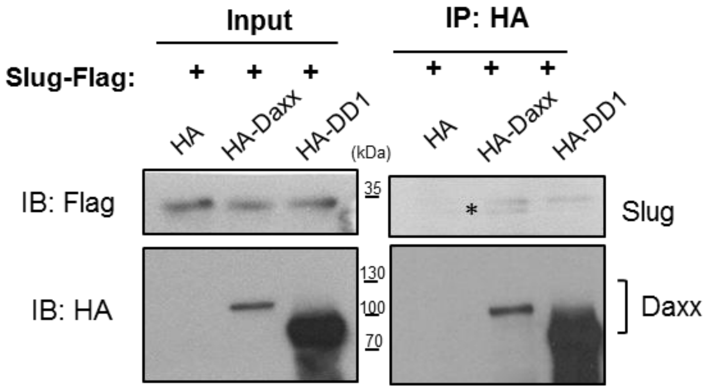

Supplementary Figure 6. Determining the role of Daxx in recruitment of HDAC1 to the promoter. H1299 cells transfected with Flag-HDAC1 were co-transfected with HA-Daxx or HA-DD1 (or empty vector) and treated with MG132 for 5 h. Cell lysates were immunoprecipitated (IP) with anti-Flag antibodies and analyzed by immunoblotting (IB).

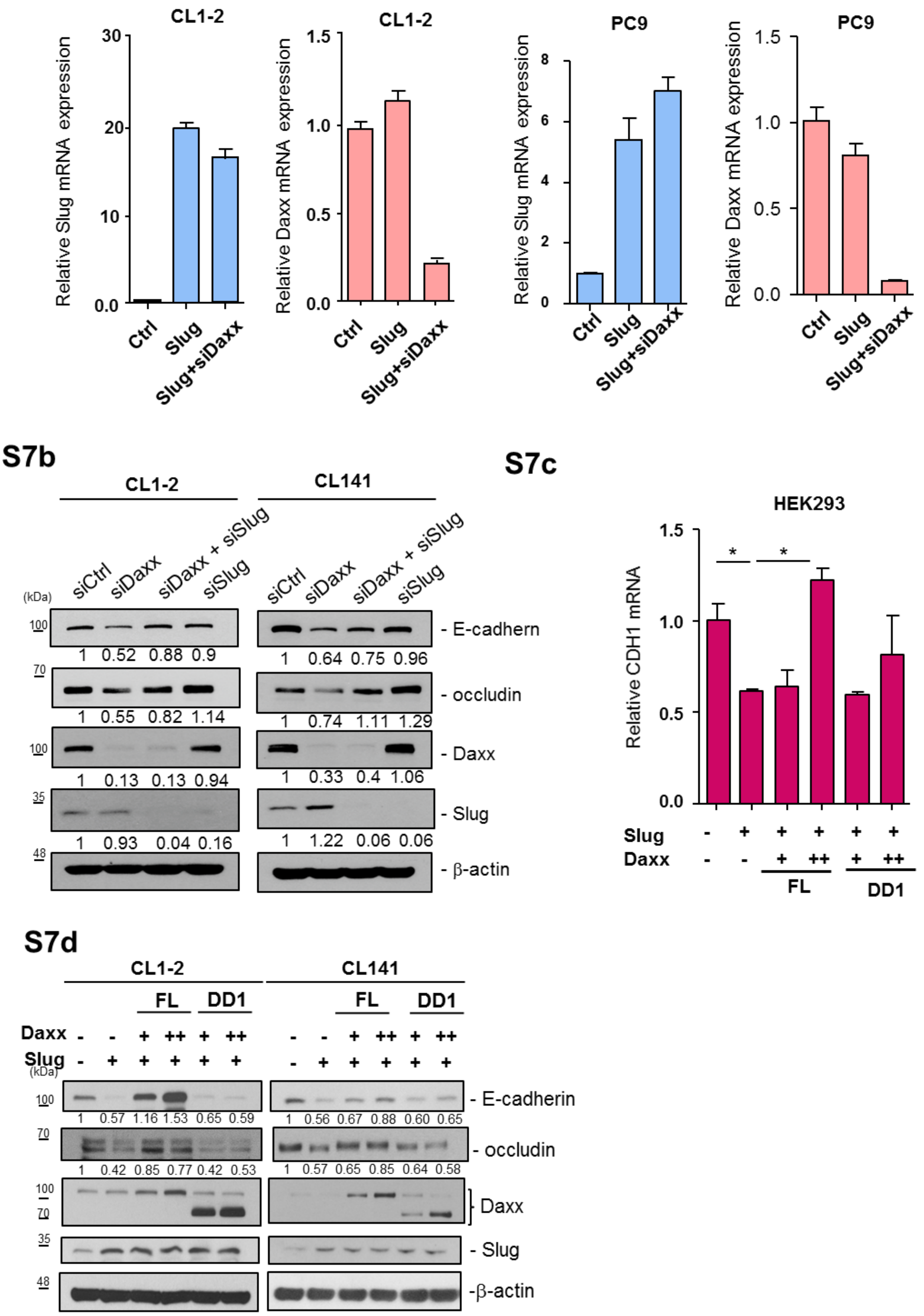

Supplementary Figure 7. Quantification of Daxx and Slug mRNA levels by real-time RT-PCR and Daxx suppresses EMT and cell invasion abilities by restraining Slug activity. (a) CL1-2 cells and PC9 cells stably expressing Neo or Slug were transfected with the indicated siRNAs and incubated for 48 h. Total RNA was isolated

for quantification of Slug and Daxx mRNA expression levels by real-time RT-PCR. Each condition was first normalized to GAPDH mRNA (internal control), and then expressed relative to normalized mRNA levels in the control group. **(b)** Double-knockdown of endogenous Daxx and/or Slug in CL1-2 and CL141 cells. Cells were subsequently analyzed by Western blotting to detect E-cadherin and occludin protein expression. **(c)** E-cadherin (CDH1) mRNA levels were analyzed in HEK-293 cells stably expressing control virus (Neo), Slug, Slug + full-length Daxx (FL) or Slug + Daxx D1 mutation (DD1), generated by infection with viral constructs at an MOI of 3. **(d)** CL1-2 and CL141 cells stably expressing control (Neo), Slug, Slug + full-length Daxx (FL), or Slug + Daxx D1 mutation (DD1) were prepared by infection with virus at a multiplicity of infection (MOI) of 1 or 3. Protein levels were determined by immunoblotting**.** Error bars represent s.d. *$P<0.05$, **$P <0.01$, paired two-way Student's t-test.

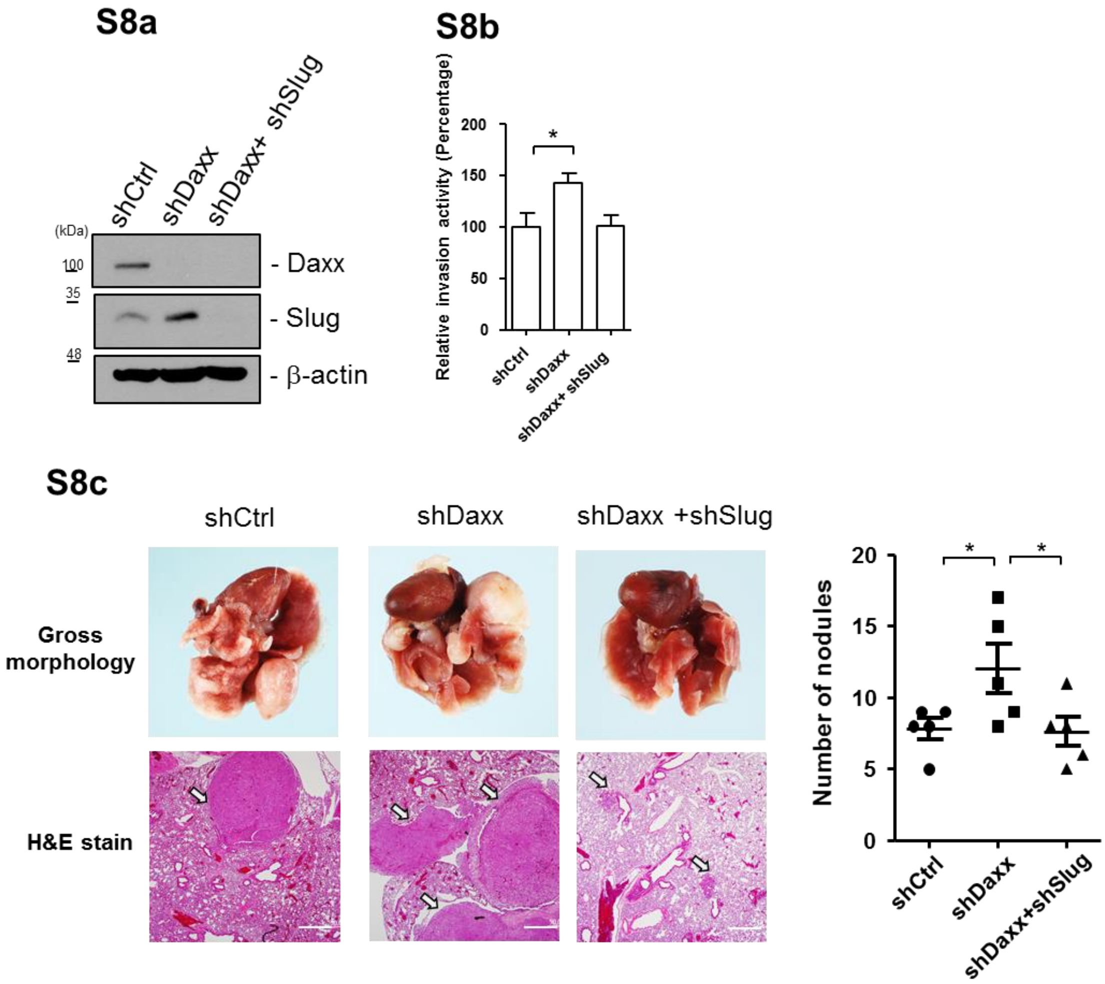

Supplementary Figure 8. Daxx negatively regulates metastasis in a Slug-dependent manner. CL1-2 stably infected with shCtrl (Trc005), shDaxx, and shDaxx+shSlug for 48 h. **(a)** The protein lysate was then subject for Western Blot. **(b)** Cells were subjected for transwell invasion assay. **(c)** Tail vein lung metastasis experiments. CL1-2 /shCtrl, CL1-2/ shDaxx, or CL1-2/shDaxx+ shSlug cells were intravenously injected into mouse tail vein to mimic extravasation of lung metastasis. Lungs of mice were harvested on day 45 after injection, and histologically examined by H&E staining and bright field microscopy. Scale bar: 50 µm. **(c, right)** Quantitative evaluation of lung metastatic nodules. Data are expressed as means ± SEM (n = 5 mice per group). Error bars represent s.d. *P<0.05, **P <0.01, paired two-way Student's t-test.

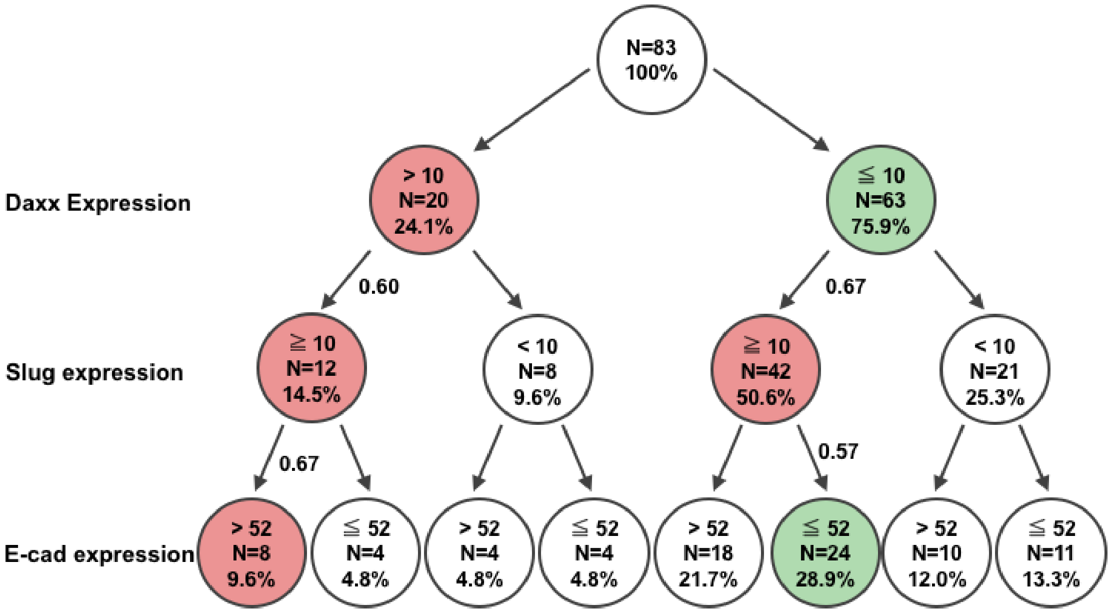

Supplementary Figure 9. The preferential expression patterns of Daxx, Slug, and E-cad represent in a tree diagram. Numbers along edges indicated conditional probabilities of choosing nodes in a path. Color red codes high expression and green codes low expression.

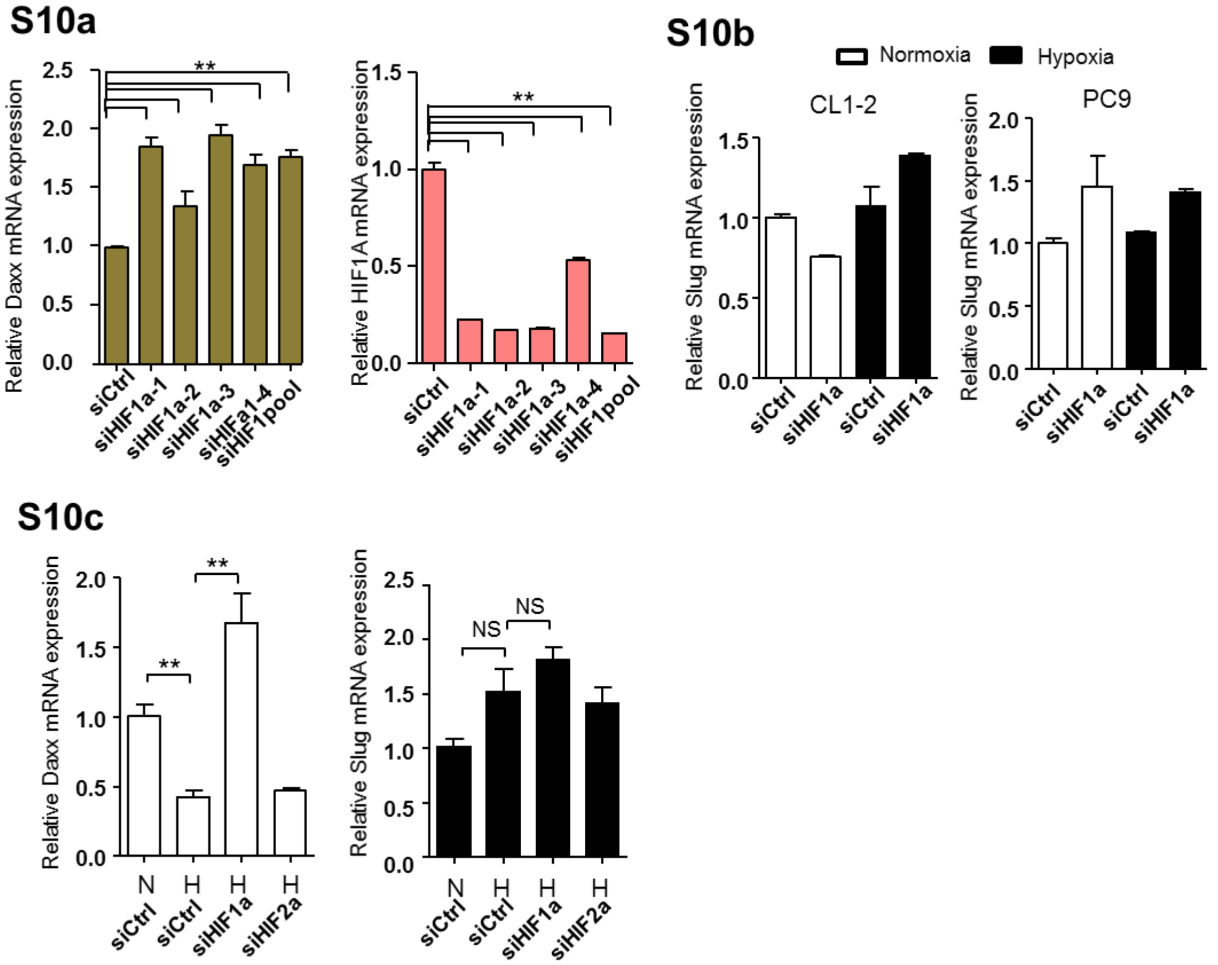

Supplementary Figure 10. Daxx is regulated by stabilized HIF-1α under hypoxic conditions. (a) Endogenous HIF-1α in CL1-2 cells was silenced using pooled siRNA (siHIF1α-pool) or individual siRNAs (siHIF1α-1–4) under hypoxic conditions. Daxx and HIF1A mRNA were then evaluated by RT-qPCR. **(b)** CL1-2 and PC9 cells were transfected with control siRNA (siCtrl) or siHIF-1α to knockdown HIF-1α, and subsequently incubated under normoxic or hypoxic conditions for 48 h. Slug mRNA expression levels was measured by real-time RT-PCR. **(c)** CL1-2 cells transfected with control siRNA (siCtrl), siHIF-1α, and siHIF-2α and incubated under normoxic (N) or hypoxic (H) conditions for 48h. Daxx and Slug mRNA then were detected by real-time RT-PCR. mRNA levels under each condition were first normalized to those of GAPDH. All data are presented as the relative ratio ± s.d. for three independent experiments**.** Error bars represent s.d., **P <0.01, NS= not significant, paired two-way Student's t-test.

S11a

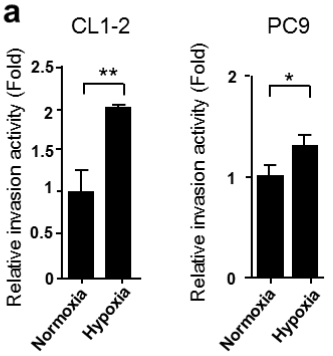

S11b

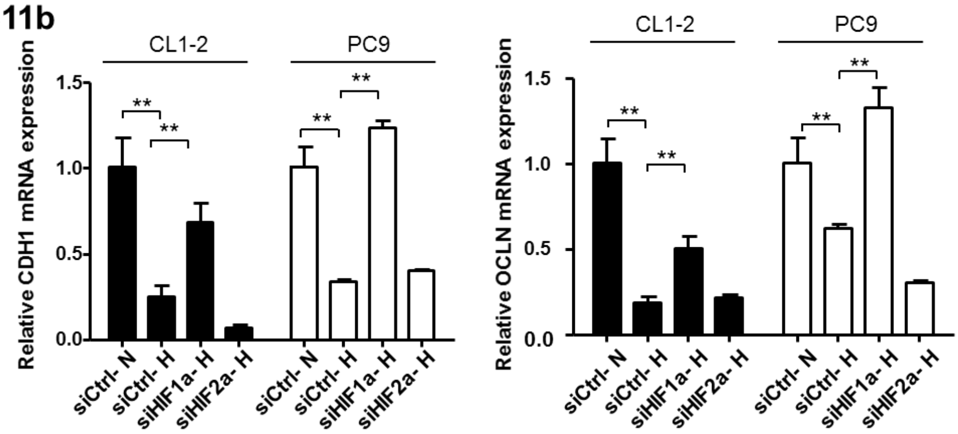

S11c

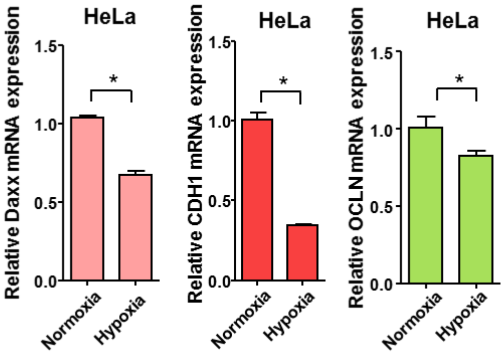

Supplementary Figure 11. Hypoxia conditions altered cell invasiveness and EMT **(a)** CL1-2 and PC9 cells were incubated under normoxic or hypoxic conditions for 24 h. Cells were subjected for invasion assay for an additional 22 h under normoxic or hypoxic conditions. The invaded cells were then harvested for counting. **(b)** CL1-2 and PC9 cells were transfected with control siRNA (siCtrl) or siHIF-1α and subsequently incubated under normoxic or hypoxic conditions for 48 h. Total RNA was isolated for quantification of E-cadherin (CDH1) and occludin (OCLN) mRNA expressions by real-time RT-PCR. CL1-2 and PC9 cells were incubated under normoxic or hypoxic conditions for 48 h. Total RNA was harvested for quantification of E-cadherin (CDH1) and occludin (OCLN) by real-time RT-PCR. **(c)** HeLa cells were incubated under normoxic (N) and hypoxic (H) conditions for 48 h. Total RNA was extracted for quantification of Daxx, E-cadherin (CDH1), and Occludin (OCLN) mRNA levels by real-

time RT-PCR. mRNA levels under each condition were first normalized to those of GAPDH, and then expressed relative to the control group. All data are presented as the relative ratio ± s.d. for three independent experiments. *$P<0.05$, **$P<0.01$, paired two-way Student's t-test.

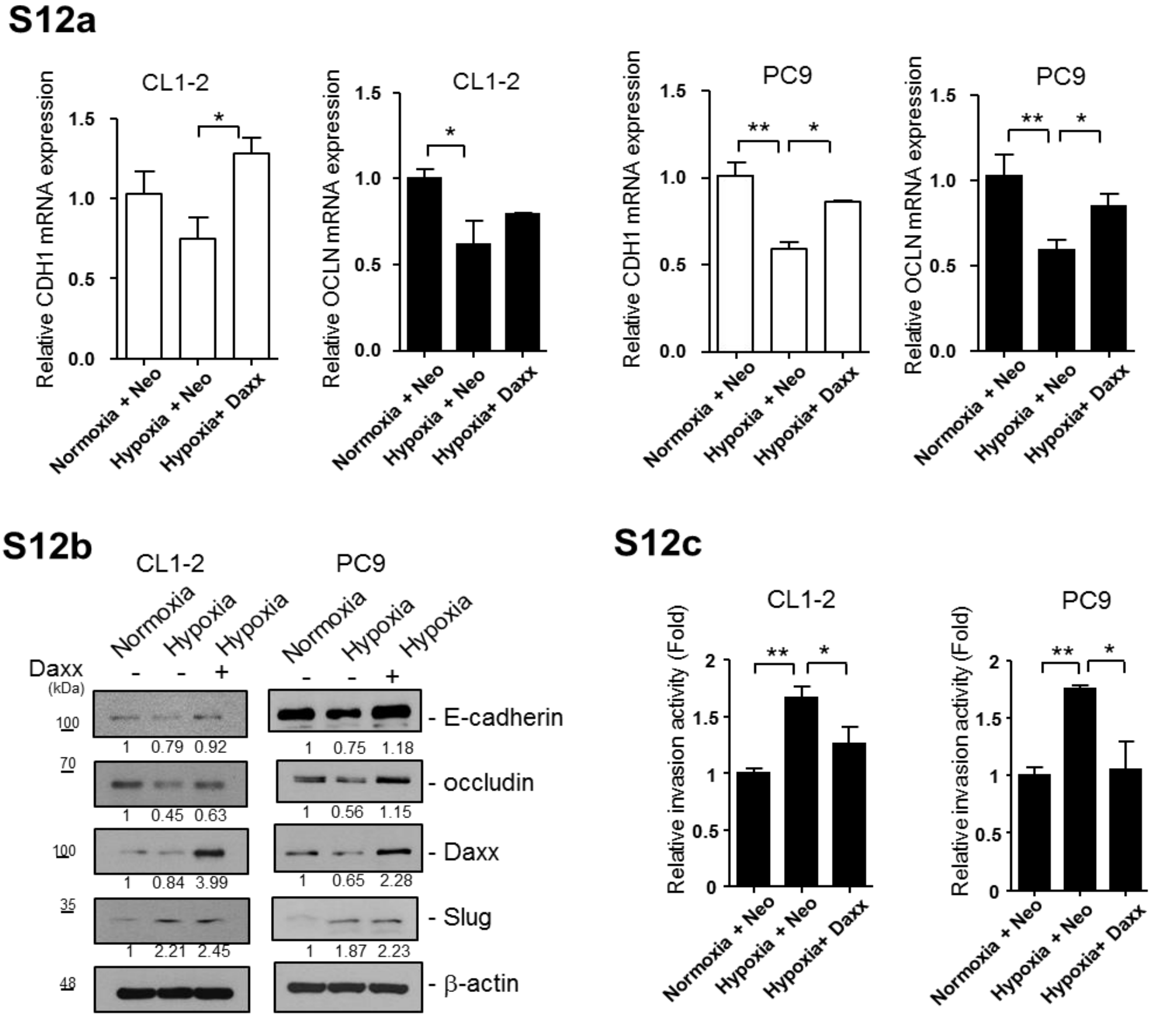

Supplementary Figure 12. Daxx re-expressing under hypoxia reversed hypoxia-mediated EMT and invasiveness. (a-c) CL1-2 and PC9 cells stably transduced with Neo or Daxx were incubated under normoxic and hypoxic conditions. **(a)**The mRNA level of E-cadherin (CDH1) and occludin (OCLN) were determined by real-time RT-PCR. **(b)** The expression of indicated proteins were determined by Western blot assay. **(c)** Invasion assays were performed under the indicated conditions. *P<0.05, **P <0.01, paired two-way Student's t-test.

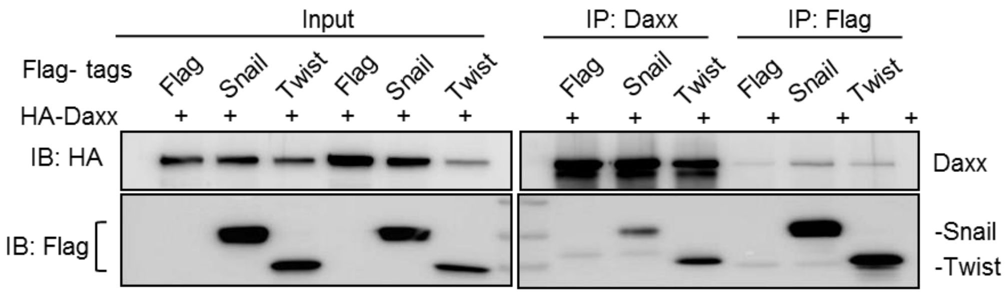

Supplementary Figure 13. Daxx interacts with Snail and Twist. H1299 cells transfected with HA-Daxx were co-transfected with Flag-Snail or Flag- Twist and treated with MG132 for 5 h. Cell lysates were immunoprecipitated (IP) with anti-Flag antibodies and analyzed by immunoblotting (IB).

Supplementary Figure 14. Original images of Western blot

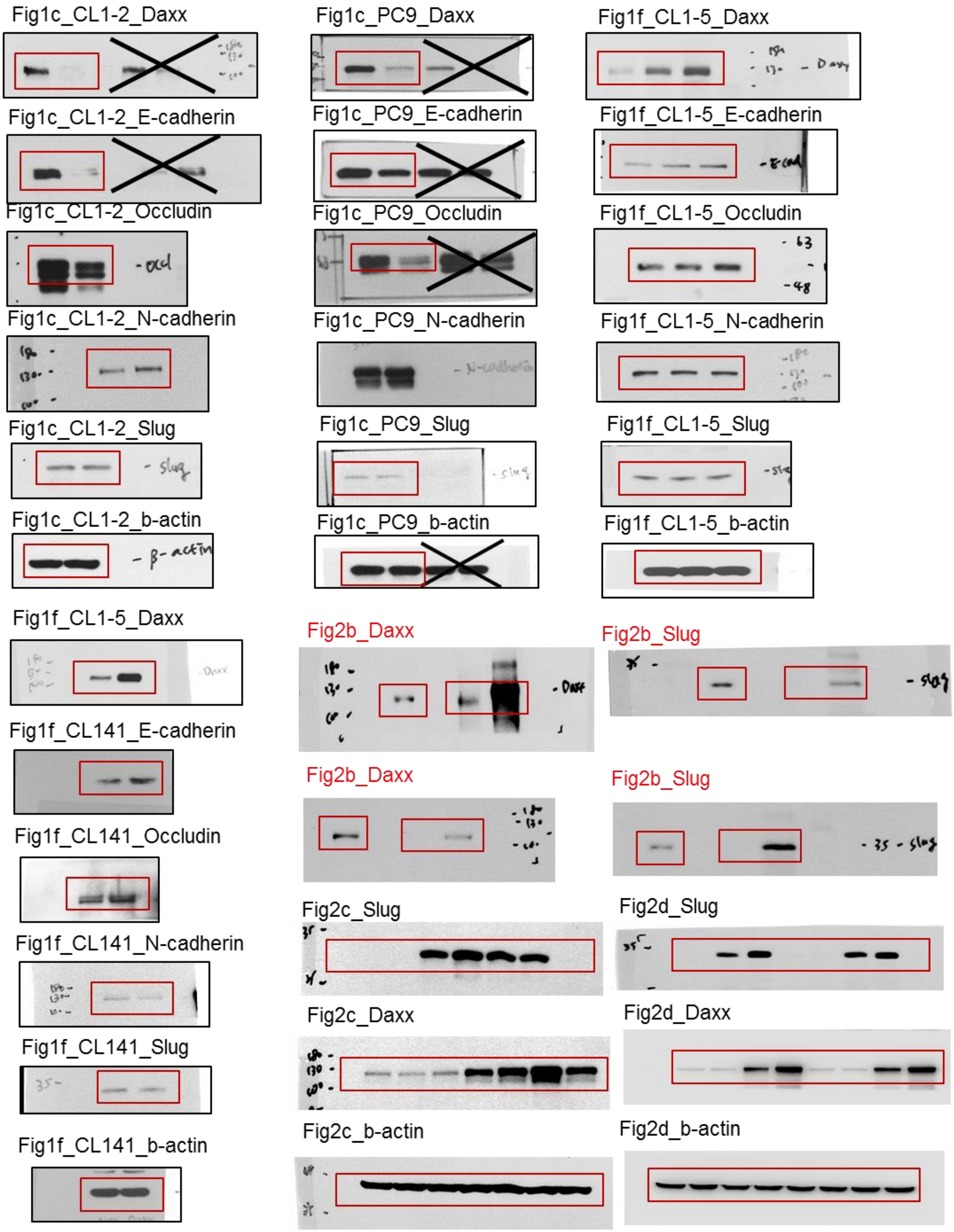

Fig3a_Input_Flag

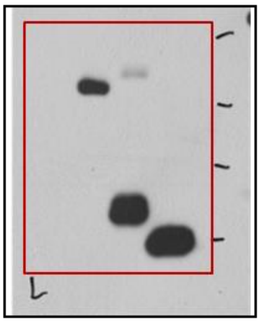

Fig3a_IP_Flag

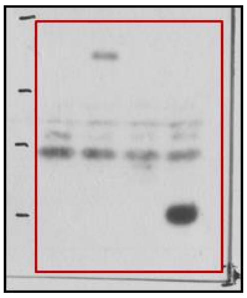

Fig3b_Input_Slug

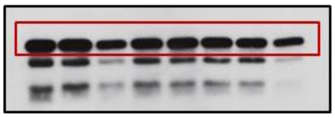

Fig3b_IP_Slug

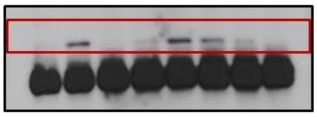

Fig3b_Input_Flag

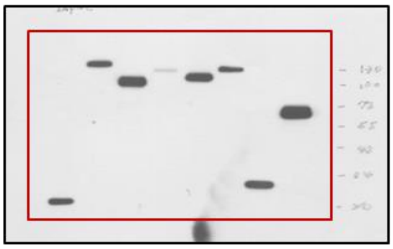

Fig3b_IP_Flag

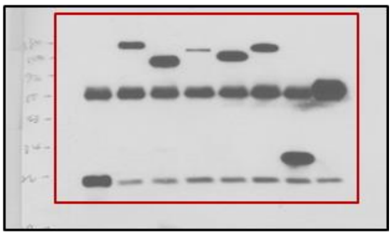

Fig3a_Input_HA

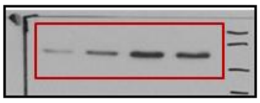

Fig3a_IP_HA

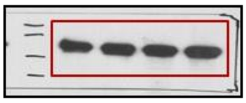

Fig3d_IP_HDAC1

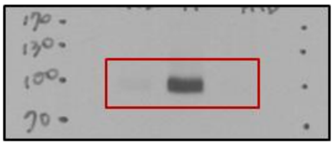

Fig3e_Input_Flag

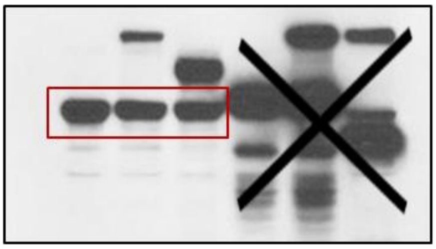

Fig3e_IP_Flag

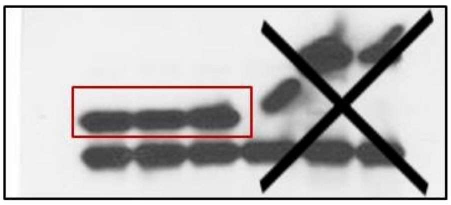

Fig3d_IP_Slug

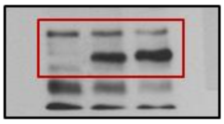

Fig3e_IP_HA

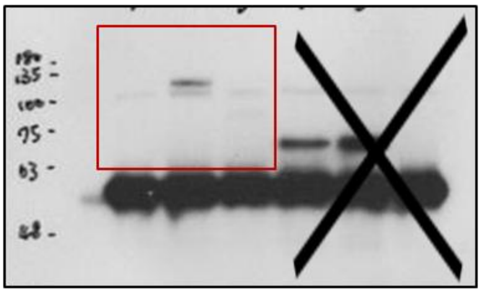

Fig3e_Input_HA

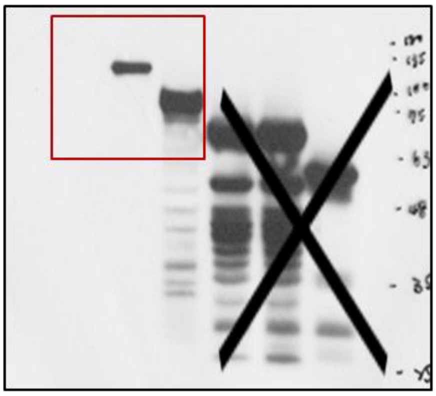

Fig3d_Input_HDAC1

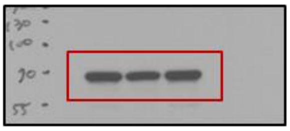

Fig3d_Input_Slug

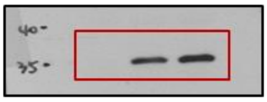

Fig7a_PC9_HIF-2α

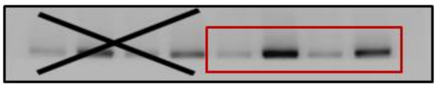

Fig3d_Input_Daxx

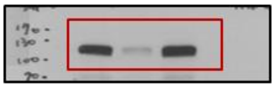

Fig7a_CL1-2_Daxx

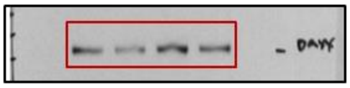

Fig7a_PC9_HIF-1β

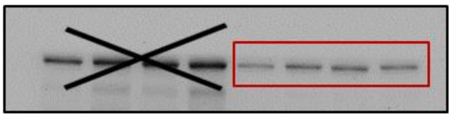

Fig7a_CL1-2_HIF-1α

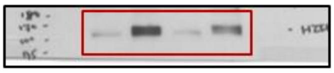

Fig7a_CL1-2_Slug

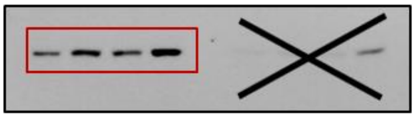

Fig7a_CL1-2_Daxx

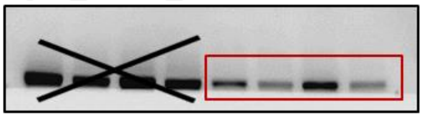

Fig7a_CL1-2_HIF-2α

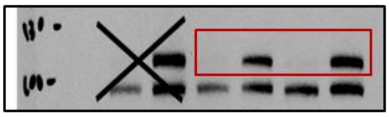

Fig7a_CL1-2_β-actin

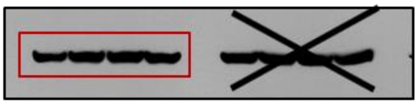

Fig7a_CL1-2_Slug

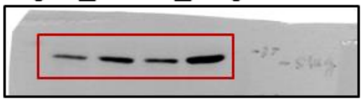

Fig7a_CL1-2_HIF-1β

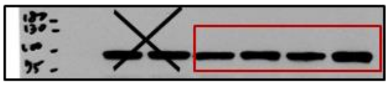

Fig7a_PC9_HIF-1α

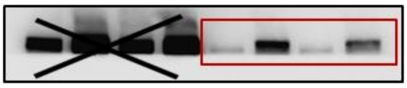

Fig7a_CL1-2_β-actin

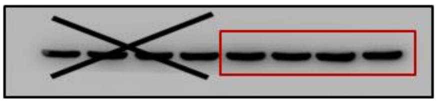

Fig7b_CL1-2_HIF-1α

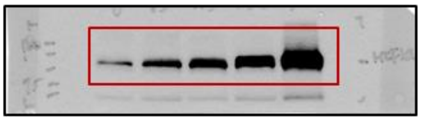

Fig7b_CL1-2_HIF-2α

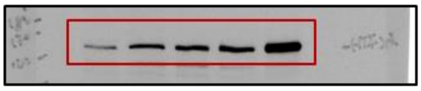

Fig7b_CL1-2_HIF-1β

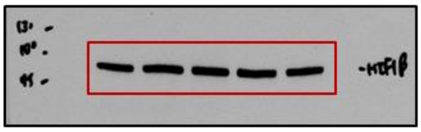

Fig7b_CL1-2_Daxx

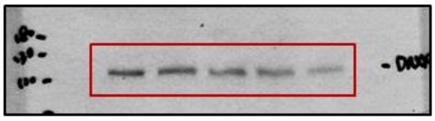

Fig7b_CL1-2_Slug

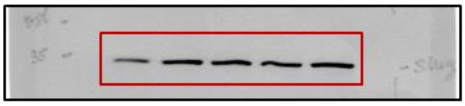

Fig7b_CL1-2_β-actin

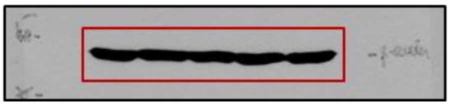

Fig7b_CL1-2_HIF-1α

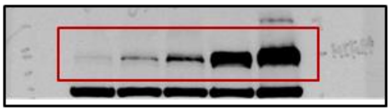

Fig7b_CL1-2_HIF-2α

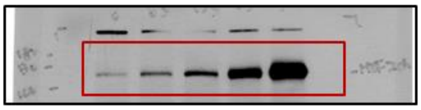

Fig7b_CL1-2_HIF-1β

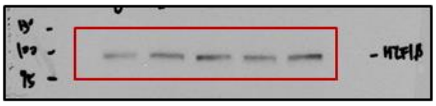

Fig7b_CL1-2_Daxx

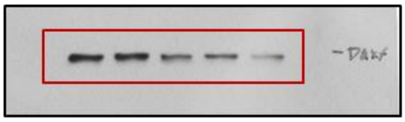

Fig7b_CL1-2_Slug

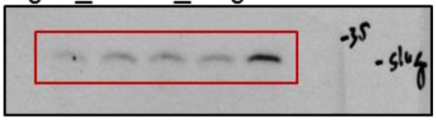

Fig7b_CL1-2_β-actin

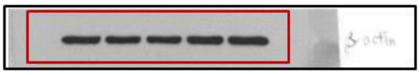

Supplementary Tables:

Supplementary Table 1. Clinical characteristics of 119 NSCLC patients with different expression levels of Slug in tumor specimens

	Total (n=119)	Slug < 10 (n=35)	Slug ≧ 10 (n=84)	P value
Age mean (SD)	63.67 (9.7)	64.06(8.85)	63.51(10.08)	0.781[a]
Gender				0.158[b]
Female	55 (64.22%)	20 (57.14%)	35 (41.67%)	
Male	64 (53.78%)	15 (42.86%)	49 (58.33%)	
Cell type				0.001[b]
BAC	3 (2.52%)	1 (2.86%)	2 (2.38%)	
Adenocarcinoma	69 (57.98%)	11 (31.43%)	58 (69.05%)	
SCC	42 (35.29%)	21 (60.00%)	21 (25.00%)	
ASC.	5 (4.2%)	2 (5.71%)	3 (3.57%)	
Smoking				0.361[b]
Non-smoker	51 (68.92%)	5 (100%)	46 (66.67%)	
Smoker	23 (31.08%)	0 (0.00%)	23 (33.33%)	
Stage				0.001[b]
II A	17 (14.29%)	11 (31.43%)	6 (7.14%)	
II B	37 (31.09%)	18 (51.43%)	19 (22.62%)	
III A	11 (9.24%)	0 (0.00%)	11 (13.1%)	
III B	28 (23.53%)	1 (2.86%)	27 (32.14%)	
IV	26 (21.85%)	5 (14.29%)	21 (25.00%)	

[a] P-value calculated using t-test, [b] P-value calculated using fisher's exact test.

BAC: bronchial adenocarcinoma carcinoma; SCC: squamous cell carcinoma; ASC: adenosquamous carcinoma

Supplementary Table 2. Slug expressions in relation to clinical parameters and pathological characteristics in 119 NSCLC

Variable	Hazard Ratio (95% CI)	P-value*
**Slug** (positive v.s negative)		
Slug	8.88 (2.06 - 38.33)	0.003
Sex	2.33 (1.21 - 4.48)	0.012
Cell type	0.51 (0.25 - 1.05)	0.066
Stage	2.06 (0.98 - 4.33)	0.058
Age	0.97 (0.94 - 1.01)	0.106

* Stepwise selection was uses to select the optimal multivariable Cox proportional hazard regression model. Slug expression was designated as 'high' or 'low' using 10% cell positivity as the cut-off point. The divided cohort was adjusted by age, sex, histological type, and stage. P-values (two-sided) were calculated using a chi-square test. CI, confidence interval.

Supplementary Table 3. Clinical characteristics of 83 NSCLC patients with different expression levels of Daxx in tumor specimens

	All (n=83)	Daxx < 10 (n=63)	Daxx ≧ 10 (n=20)	P value
Age mean (SD)	63.51 (10.14)			0.65[a]
Gender				0.60[b]
Male	48 (57.83%)	35 (55.56%)	13 (65.00%)	
Female	35 (42.17%)	28 (44.44%)	7 (35.00%)	
Cell type				0.07[b]
BAC	2 (2.41%)	0 (0.00%)	2 (10.00%)	
Adenocarcinoma	57 (68.67%)	46 (73.02%)	11 (55.00%)	
SCC	21 (25.30%)	15 (23.81%)	6 (30.00%)	
ASC.	3 (3.61%)	2 (3.17%)	1 (5.00%)	
Smoking				0.78[b]
Non-smoker	45 (66.18%)	33 (67.35%)	12 (63.16%)	
Smoker	23 (33.82%)	16 (32.65%)	7 (36.84%)	
Stage				0.73[b]
II A	6 (7.23%)	4 (6.35%)	2 (10.00%)	
II B	19 (22.89%)	15 (23.81%)	4 (20.00%)	
III A	11 (13.25%)	10 (15.87%)	1 (5.00%)	
III B	27 (32.53%)	20 (31.75%)	7 (35.00%)	
IV	20 (24.10%)	14 (22.22%)	6 (30.00%)	

[a] P-value calculated using t-test, [b] P-value calculated using fisher's exact test.

BAC: bronchial adenocarcinoma carcinoma; SCC: squamous cell carcinoma; ASC: adenosquamous carcinoma

Supplementary Table 4. Daxx, Slug, or/ and E-cadherin expressions in relation to clinical parameters and pathological characteristics in 83 NSCLC

Variable	Hazard Ratio (95% CI)	P-value*
**Daxx(>10% v.s ⩽10%)**		
Daxx	0.202 (0.06- 0.681)	0.0099
Sex	1.59 (0.609-4.15)	0.3435
Cell type	1.282 (0.586-2.805)	0.5339
Stage	1.089 (0.732-1.62)	0.6749
Age	0.989 (0.95-1.03)	0.5896
**E-cadherin (>52 % v.s. ⩽52 %)**		
E-cadherin	0.64 (0.32- 1.25)	0.19
Sex	1.86 (0.94-3.68)	0.076
Cell type	0.59 (0.28- 1.25)	0.172
Stage	1.76 (0.79- 3.95)	0.167
Age	0.98 (0.95- 1.25)	0.272
**Combine (Daxx^{+}/ Slug$^{high/ low}$ v.s Daxx^{-}/ Slug^{high} v.s Daxx^{-}/ Slug^{low})**		
Daxx^{+}/ Slug$^{high/ low}$	0.26 (0.07 - 0.99)	0.048
Daxx^{-}/ Slug^{high}	1.94 (0.86 - 4.38)	0.11
Sex	2.60 (1.33 - 5.11)	0.005
Cell type	0.66 (0.31 - 1.44)	0.301
Stage	2.24 (1.03 - 4.87)	0.041
Age	0.98 (0.95 - 1.02)	0.347
**Combine (Daxx^{+}/E-cad^{high} v.s Daxx^{-}/ E-cad^{low} v.s Others)**		
Daxx^{+}/ E-cad ^{high}	0.20 (0.05 - 0.86)	0.031
Others	0.35 (0.17 - 0.72)	0.004
Age	0.99 (0.98 - 1.03)	0.749
Sex	2.00 (0.32 - 3.92)	0.042
Cell type	0.67 (0.32 - 1.41)	0.289
Stage	1.64 (0.75 - 3.58)	0.217

* Stepwise selection was uses to select the optimal multivariable Cox proportional hazard regression model. Daxx expression was designated as ‘high’ or ‘low’ using 10% cell positivity as the cut-off point, Slug cut-off using 10% nucleus positivity, while E-cadherin cut-off point was set as 52% positive expression. The divided cohort was adjusted by age, sex, histological type, and stage. P-values (two-sided) were calculated using a chi-square test. CI, confidence interval.

Supplementary Table 5. HR for death among 226 lung adenocarcinoma patients according to multivariable Cox regression assay

Variable	Hazard ratio (95% C.I.)	P value
SCORE	0.48 (0.3 - 0.75)	0.001
stage	2.89 (1.44 - 5.81)	0.003
age	1.03 (0.98 -1.07)	0.23

SCORE (Hypoxia low: 3; Hypoxia high/ Daxx high: 2; Hypoxia high/ Daxx low : 1)

Supplementary Table 6. Relationship between EGFR mutation status and the protein expression of Daxx, Slug, and E-cadehrin in 77 NSCLC specimens.

		EGFR mutation status		
		Positive (n=37)	Negative(n=39)	P value*
**Daxx**	low	29 (50%)	29 (50%)	0.7896
	high	8 (44.44%)	10 (55.56%)	
**Slug**	low	9 (34.62%)	17 (65.38%)	0.094
	high	28 (56%)	22 (44%)	
**E-cadherin**	low	15 (39.47%)	23 (60.53%)	0.1681
	high	22 (57.89%)	16 (42.11%)	
**Daxx/ E-cadherin**	Daxx-/ E-cad-	14 (45.16%)	17 (54.84%)	0.6073
	Daxx+/ E-cad +	7 (63.64%)	4 (36.36%)	
	Others	16 (47.06%)	18 (52.94%)	

*P-values were calculated by Fisher's Exact Test

Supplementary Table 7. Listed siRNA sequences

siSlug-pool	GGACACACAUACAGUGAUU
	UAAAUACUGUGACAAGGAA
	GAAUGUCUCUCCUGCACAA
	GAAUCUGGCUGCUGUGUAG
siDaxx-pool	CUACAGAUCUCCAAUGAAA
	GCUACAAGCUGGAGAAUGA
	GGAAACAGCUAUGUGGAAA
	GGAGUUGGAUCUCUCAGAA
siHIF1A-pool	GGACACAGAUUUAGACUUG
	GAUGGAAGCACUAGACAAA
	CGUGUUAUCUGUCGCUUUG
	GAUGAAAGAAUUACCGAAU
siOCLN-pool	GAAGAAAGAUGGACAGGUA
	GUACAUGGCUGCUGCUGAU
	AUAAAGAACUCUCCCGUUU
	AAGUGAAUGACAAGCGGUU

Supplementary Methods:

Expression of GST fusion proteins and GST pull-down assay

Recombinant GST-Slug expression was induced in the bacterial strain XA-90 by incubating with 0.5 mM isopropyl β-D-1-thiogalactopyranoside (IPTG) for 30 min and then purified with glutathione beads (GE Healthcare/Amersham Biosciences, Buckinghamshire, UK) according to the standard protocol. HA-Daxx was produced using TNT Quick Coupled Transcription/Translation Systems (Promega, WI, USA). HA-Daxx was mixed with purified GST-Slug or GST alone. Pull-down assays were performed at 4°C for 1 h. After thoroughly washing beads with phosphate-buffered saline (PBS), bound proteins were eluted by boiling in SDS sample buffer, separated by SDS-PAGE, and detected by immunoblotting.
